# Developmental Changes in Morphology of the Middle and Posterior External Cranial Base in Modern *Homo sapiens*

**DOI:** 10.1155/2015/324702

**Published:** 2015-08-27

**Authors:** Deepal H. Dalal, Heather F. Smith

**Affiliations:** ^1^Department of Biomedical Sciences, Midwestern University, Glendale, AZ 85308, USA; ^2^Department of Anatomy, Midwestern University, Glendale, AZ 85308, USA; ^3^School of Human Evolution and Social Change, Arizona State University, USA

## Abstract

The basicranium has been described as phylogenetically informative, developmentally stable, and minimally affected by external factors and consequently plays an important role in cranial size and shape in subadult humans. Here basicranial variation of subadults from several modern human populations was investigated and the impact of genetic relatedness on basicranial morphological similarities was investigated. Three-dimensional landmark data were digitized from subadult basicrania from seven populations. Published molecular data on short tandem repeats were statistically compared to morphological data from three ontogenetic stages. Basicranial and temporal bone morphology both reflect genetic distances in childhood and adolescence (5–18 years), but not in infancy (<5 years). The occipital bone reflects genetic distances only in adolescence (13–18 years). The sphenoid bone does not reflect genetic distances at any ontogenetic stage but was the most diagnostic region evaluated, resulting in high rates of correct classification among populations. These results suggest that the ontogenetic processes driving basicranial development are complex and cannot be succinctly summarized across populations or basicranial regions. However, the fact that certain regions reflect genetic distances suggests that the morphology of these regions may be useful in reconstructing population history in specimens for which direct DNA evidence is unavailable, such as archaeological sites.

## 1. Introduction

Cranial morphology is frequently studied with the purpose of identifying and interpreting the extensive range of variation that exists among modern human populations and in the hominin fossil record (e.g., [1–10]). The cranium is a valuable structure for studying the genetic and ontogenetic relationships among* Homo sapiens* based on geographic provenance, population affinities, and dietary proclivities [1–7]. The vast majority of studies on the relationship between cranial morphology and genetic relatedness, however, have focused on adult crania. To date, little data exist on the ontogenetic trajectories of the developing cranium, especially the basicranium, as they relate to molecular distances among populations. Determining how basicranial shape develops in infants, juveniles, and young adults has implications for bioarchaeology and paleoanthropology, in which skeletal specimens are often discovered that contain no DNA and thus no direct genetic evidence of ancestry. In particular, there would be great utility for future bioarchaeological studies to determine whether the basicranium can be used to classify isolated subadult specimens in populations and draw conclusions regarding the ancestry or population history from basicranial shape of a subadult specimen or group of specimens.

Previous studies have demonstrated that the basicranium is a phylogenetically informative region among human and nonhuman primate adults [11–15]. Human populations differ significantly in their adult basicranial morphology, and these differences reflect the genetic relatedness among populations [1, 3, 4, 6]. The basicranium begins to ossify early in the prenatal period, at approximately 11 to 12 weeks [16–18], experiences minimal strain [19–23], and is less susceptible to external factors, such as environment and diet, than other regions of the cranium [2, 3, 15]. It is also developmentally stable [11, 13, 14, 24, 25], because it emerges from a cartilaginous template early in fetal life, making this region less susceptible to nongenetic forces during ontogeny than the externally sensitive intramembranous bones of the facial skeleton [19, 20]. Additionally, the osseous morphology of the basicranium experiences a lower degree of masticatory stresses than other cranial modules, such as the splanchnocranium (face). The higher strain regions are consequently more subject to biomechanical stress and therefore exhibit higher levels of variability [26]. As such, it has been argued that low strain cranial regions, such as the basicranium, should be more stable and more phylogenetically informative than higher strain regions, such as the face [26]. However, it should be noted that this prediction has not held up to empirical testing in several hominoid (ape) species [5, 26–28].

Nonetheless, since the cranium starts to develop before birth [16–18] and continues to develop until adulthood, it is fundamental to understand the ontogenetic processes, including a comparison of these processes in diverse groups of humans. This can lead to a thorough understanding of how the basicranium develops and whether it reflects among population genetic distances at various stages of subadult ontogeny.

Previous studies have compared the shape of the developing facial skeleton among groups from different geographic locations and genetic backgrounds to determine whether similar ontogenetic processes characterize divergent groups [7–10]. A wide range of variation in human craniofacial form exists and can be relatively easily altered through minor shifts in the ontogenetic process [9]. There have been a limited number of previous studies comparing cranial ontogenetic patterns among human populations, and most have been limited to the temporal bone [7, 29–31]. Thus, the morphological patterning of human cranial ontogeny is still relatively poorly understood.

To date, there have been extensive studies on the morphology of the temporal bone and its applications to phylogeny [32–34], evolution, and ontogeny [7]. The temporal bone demonstrates genetic and geographic patterning that is consistent with a predominantly neutral evolutionary history, shaped primarily by mutation, genetic drift, and gene flow [1–7, 34]. However, despite the abundance of studies on temporal bone shape, there is comparatively little information on the ontogenetic trajectories that result in morphological variation at various subadult stages of development.

Modern human populations can be readily distinguished from one another based on one particular component of the basicranium and temporal bone shape [2–4, 34]. More recently, Smith and colleagues [7] compared subadult and adult temporal bone morphologies in modern* Homo sapiens* and found that significant differences among populations originate early in ontogeny. Specifically, individuals from different populations can be differentiated based on temporal bone shape prior to the eruption of the first molar [7]. Subsequent developmental stages progress in a relatively similar manner with essentially parallel ontogenetic trajectories, however, maintaining those original differences into adulthood [7]. These findings are limited to the temporal bone but encourage further analysis of other cranial regions, including other larger regions, such as the basicranium, in relation to genetics, geography, and population differences.

Several researchers have attempted to determine whether the basicranium is a more reliable region for reconstructing genetic distances than other regions of the cranium [2, 3, 32]. Smith [3] found that the basicranium was significantly more highly correlated with molecular distances than the cranial vault. Morphology of the temporal bone and the upper face were also correlated with a molecular distance matrix, demonstrating that these bones are phylogenetically informative. von Cramon-Taubadel concluded, however, that the basicranium is not significantly more congruent with genetic data than the intramembraneously ossifying cranial modules (i.e., cranial vault and face) [5, 36]. In sum, the basicranium “is a good place to look for reliable characters…that describe developmental processes or events” [13, page 159], but its phylogenetic utility during various stage of development is minimally understood.

In light of the previous work and unresolved research questions outlined above, the aims of this study are to determine at what point during the ontogenetic process population-specific basicranial morphologies emerge and to compare basicranial ontogeny among human populations. Specifically, we test the following hypotheses:

(H1) Human populations differ significantly in the shape of the basicranium and its various components irrespective of ontogenetic stage.
(H2) Differences among human populations in basicranial morphology are significantly correlated with their genetic distances throughout ontogeny.

## 2. Methodology

### 2.1. Data Collection

Three-dimensional data on basicranial morphology were collected by one of us (DHD). The data were collected from skulls of seven modern human populations at various ontogenetic stages (Table 1) from the American Museum of Natural History, New York, NY, and The National Museum of Natural History at the Smithsonian Institute, Washington, DC. These populations were chosen based on a single primary criterion: having sufficient cranial ontogenetic series available in museum collections. This criterion is a limiting factor, because museum collections typically consist of primarily adult skulls, since fewer individuals die as subadults and end up housed in collections. Even for individuals that die young, the smaller size and more delicate nature of their crania lead to taphonomic processes being more destructive to subadult skulls, and even those that end up in collections are more likely to be too damaged and fragmentary to measure. Thus, the populations chosen for inclusion in this study were primarily determined by the availability of well-preserved subadult cranial material. Additionally, for the sake of comparability, these populations were identical to the seven populations included in a previous study that we conducted on human temporal bone ontogeny [7].

Prior to traveling to the museums, an intraobserver error study was conducted to ensure the accuracy of the data collection. Two adult crania were digitized ten times each. To minimize the effect of investigator fatigue, the data were collected on two separate days. A paired samples* t*-test was performed, comparing the Procrustes residuals from the different trials on each skull. The paired* t*-test did not yield significant differences (*p* value = 0.96 to 0.99), indicating that the individual landmarks were consistently measured without significant error.

**Figure 1:**
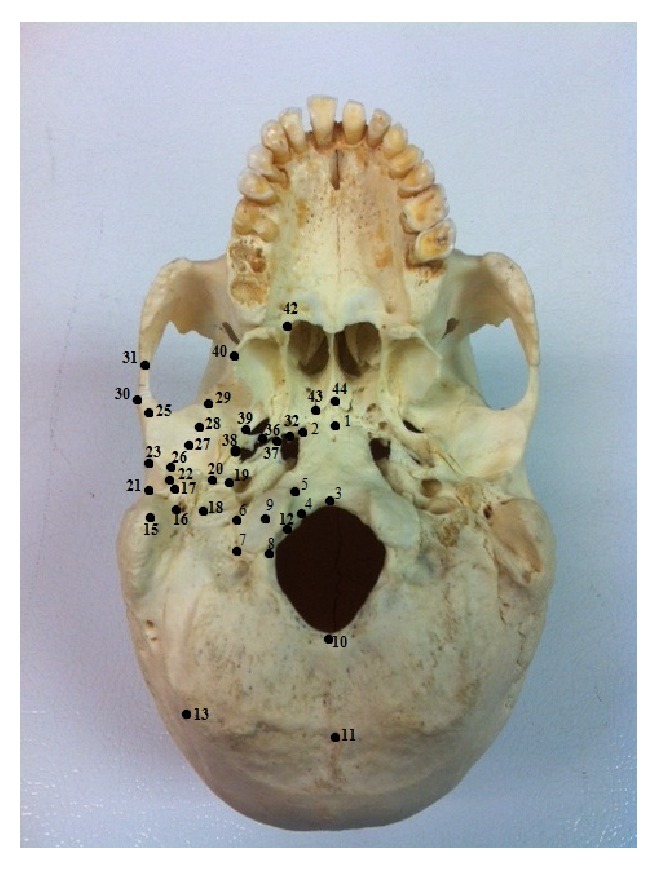
Forty-four landmarks of the basicranium digitized in the present study. Please refer to Tables 2(a)–2(c) for landmark descriptions.

Forty-four landmarks were collected from the basicranium and subcategorized into temporal, occipital, and sphenoidal regions (Tables 2(a)–2(c); Figure 1). These cranial regions have been historically underrepresented in traditional craniometric studies (e.g., [37]). Thus, in order to sufficiently capture the morphology of these regions, it was necessary to include a few empirical, instrumentally determined landmarks in addition to the traditional craniometric points (Tables 2(a)–2(c)). Due to the fact that subadult specimens are more vulnerable to taphonomic processes and often fragmentary, not all subadult specimens here were complete for all cranial regions evaluated, and thus not all specimens were included in all analyses. These landmark data were collected using a MicroScribe G2 digitizer (Immersion Corp., San Jose). Following Smith et al. [7], subadult specimens were assigned a developmental age estimate based on established dental eruption standards [37]: Age category 1 (AC1) = M1s not yet erupted; Age category 2 (AC2) = M1s erupted but not M2s; Age category 3 (AC3) = M2s erupted but not M3s [33]. In humans, these stages correspond roughly to chronological ages of <5 years, 5–12 years, and 13–18 years [37–39]. A sample of adult specimens from each population was also digitized; however, since it is not possible to accurately estimate ages for adult crania, these specimens were included in only a subset of the analyses (explained in further detail below).

Data on individual genotypes for 783 short tandem repeats (STRs) were compiled from Ramachandran et al. [40, 41] and Rosenberg et al. [35, 42] for molecular population representatives matching as closely as possible the populations from which the morphological data were collected. This practice of matching morphological and molecular populations has been successfully employed by previous studies [1–7, 34, 36]. Some of our morphological populations (i.e., the Alaskan and Egyptian samples) were not included in the Rosenberg studies from which the molecular data were derived, and thus, STR data were not available for these populations. In these cases, we attempted to choose a molecular population representative that was as similar as possible to the morphological sample. For the Alaskan sample, we chose the Yakut (a Siberian native population), and for the Egyptian sample we chose the Mozabite (an Algerian Berber group). We recognize that these imperfectly matched molecular representatives introduce a certain degree of incompatibility between the two data types; however, this mismatch renders any correlations that we obtain between the morphological and molecular distances to be a conservative, minimum estimate of the relationships between these types of data.

STRs are composed of back-to-back repeating segments of two to six nucleotides and are found at many locations within the genome [38, 39]. They are often variable in their number of repeats, and the lengths of the STRs differ among different populations, in patterns that reflect their ancestry and relatedness. STRs are appropriate for assessing human population distances because they evolve primarily neutrally and are homologous [35, 40–43]. They are also well-typed for a large number of human populations from geographically diverse locations, cultural backgrounds, and linguistic traditions [36, 37].

### 2.2. Analytical Methods

The morphological data were analyzed using a Generalized Procrustes Analysis (GPA), in which the digitized points were rotated and translated and specimens were scaled to the same size, such that the only remaining differences among them were directly attributable to shape. The GPA was followed by a Principal Components Analysis (PCA) using MorphoJ [44]. Adult specimens were included in the PCA for the purpose of visualizing how basicranial shape varied across age categories and among populations. Procrustes distances among populations were then calculated in MorphoJ for the individual subadult age categories, while further separating the data sets into basicranial, temporal, sphenoidal, and occipital regions. Each set of resulting pairwise population distances was entered into distance matrices for each cranial dataset. The significance of these population differences was assessed using a permutation test of 1000 replicates for each region.

In order to assess the degree to which basicranial morphology can be utilized to correctly classify individuals of various ages into the population from which they derived, a discriminant function analysis (DFA) was conducted using the Principal Component (PC) scores from the PCA. The DFA was used to determine whether groups could be classified reliably or if there was excessive morphological overlap. This analysis was conducted for the morphology of the basicranium and then for each of its major components, the temporal, occipital, and sphenoidal regions. These tests were conducted with cross-validation using SPSS version 11.0.1 (SPSS, Chicago, IL). This analysis indicated how well basicranial shape discriminates among populations at the three subadult stages of ontogeny (AC1, AC2, AC3).

A matrix of Slatkin's molecular distances among the molecular representative of these populations was calculated from the published molecular data using Arlequin 3.11 [45] (Table 3). The matrix of pairwise population Procrustes distances for each cranial region was then statistically compared to the molecular distance matrix using Mantel tests [46] to statistically assess and quantify the correlation among matrices. This analysis indicated whether the morphology of the basicranium, and the regions contained therein, reflected genetic distances at each developmental stage.

A series of descriptive analyses were conducted to evaluate the ontogenetic trajectory in the sample. Principal Component scores were regressed against centroid size and biological age (as determined using dental eruption of each specimen, following Ubelaker [37]) to determine the PCs that were significantly correlated with size or age and therefore indicative of age-related changes in basicranial shape. The regression of centroid size and age against PC scores reveals how shape changes with age (ontogeny) and size (allometry) [8]. Finally, a wireframe was constructed by connecting points in shape-space and morphing them along each major PC axis in Morphologika 2 [47], for the PCs that were found to explain >5% of the variance. This process allowed us to visualize shape changes along these PC axes in the different populations and age categories and describe how basicranial shape changes across all populations and age categories.

## 3. Results

### 3.1. Differentiation and Classification of Populations

Procrustes distances based on basicranial morphology of populations sampled in this study were found to be statistically significant for all combined-age subadult samples (Table 4). When separated into age categories, the majority of the Procrustes distances were significantly different among populations. There were no consistent patterns of population differences among the age categories or regions of the basicranium. The Procrustes distances showed a higher number of pairwise significant differences among populations as the age of the individuals and sample sizes increased. Hence, at AC1, given the smaller sample size for some regions (especially the unfused occipital bone), most populations were not significantly different from each other (Table 5). In AC2, more significant differences were found (Table 6), increasing in AC3 (Table 7). Therefore, (H1)—that human populations differ in basicranial shape at every ontogenetic stage—was not supported.

DFAs were performed on all regions of the basicranium: temporal, occipital, and sphenoidal. The DFA for the entire basicranium resulted in cross-validated classification rates ranging from 13.3–34.8% (Table 8). The individual cranial regions yielded higher average correct classification rates: temporal = 41.3%, occipital = 42.6%, and sphenoid = 50.0% (Tables 6–8).

The Egyptian population was one of the most correctly classified in the basicranial (33.3%), temporal bone (55%), and occipital bone (35.7%) data sets (Tables 8–11). The Egyptian population was the most correctly classified population in all regions of the basicranium except the sphenoid bone (Tables 8–11). As predicted, the three closely related Native American populations (Mexican, Utahan, and Peruvian) were frequently classified as each other. Consequently, the least correctly classified group overall was the Utah population.

### 3.2. Correspondence between Genetic Distances and Basicranial Morphology

The morphological Procrustes distances based on the different age categories and regions of the basicranium were statistically compared to the molecular *F*_ST_ distances based on STR data using Mantel tests. In the combined subadult sample, the morphology of the basicranium and occipital bone was significantly correlated with molecular distances (Table 12). Following the analysis with all populations, populations were systemically removed from the analyses to determine whether the results were unduly influenced by the inclusion of any particular population. When the Egyptian population was excluded from the analyses, the correlations between morphology and molecular distance increased to the point of significance in several additional subgroups (Table 13). Results from both analyses are reported in further detail below.

When the subadult samples for each cranial region were divided into separate age categories, more distinct patterns emerged (Tables 12 and 13). Morphology in AC3 individuals was significantly correlated with genetic distances for the basicranium, temporal, and occipital regions (Table 13). In AC2 specimens, morphology of the basicranium and temporal bone was correlated with the genetic matrix (Table 13). There were no significant correlations between the morphology of any cranial region and genetic distances for AC1 (Table 13); however, the sample sizes of these datasets were much smaller than the other age categories, rendering these results more tenuous.

Overall, the above patterns show that the shape of the basicranium, temporal bone, and occipital bone, for all populations excluding the Egyptian population, reflects genetic distances for the combined subadult sample and AC3 (13–18 years of age), thus supporting (H2) (basicranial differences are correlated with genetic distances) for these data sets. The temporal bone and basicranium also reflect genetic distances during AC2 (5–12 years of age) when the Egyptian population is excluded. The morphology of the sphenoid bone does not reflect genetic distances for subadults of any age category; however, its morphology can be used to discriminate among populations with slightly higher rates than the other two cranial regions. Therefore, (H2) is not supported for the sphenoid, AC1 (for any morphological region), or the occipital bone for AC2.

### 3.3. Ontogenetic Trajectories

#### 3.3.1. Age

**Figure 2:**
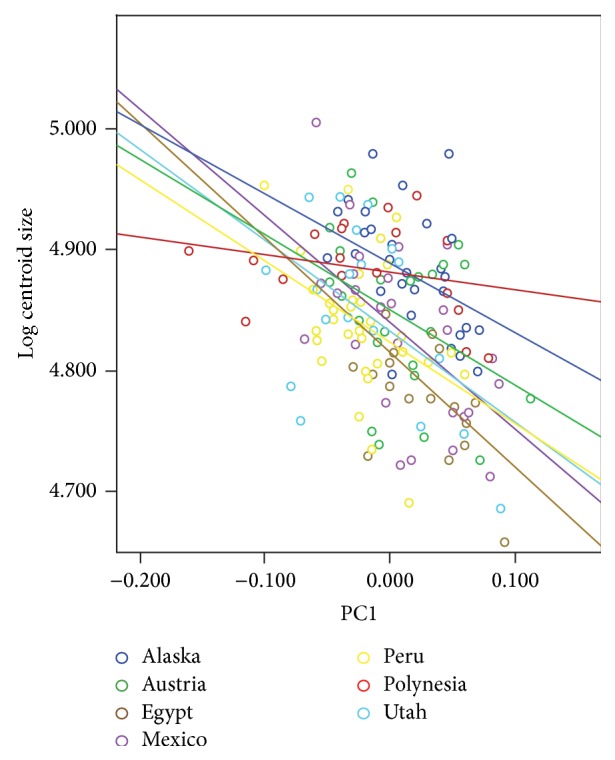
Regression plot of PC1 scores versus log centroid size for the basicranium. The individual population regression lines are indicated and their* R* ^2^ values indicated.

**Figure 3:**
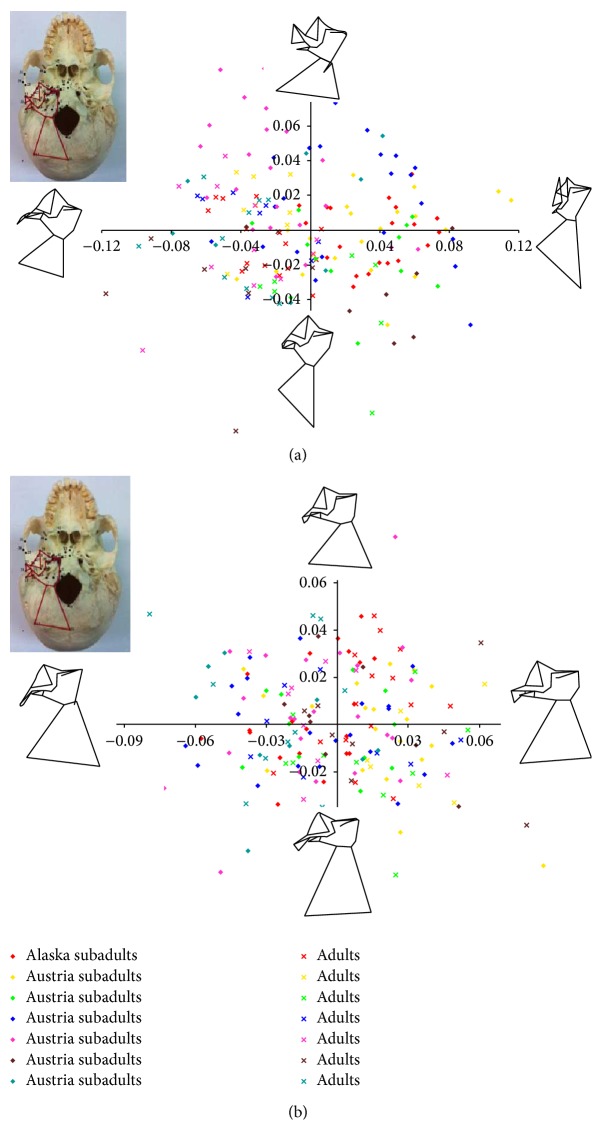
Plot of the first four principal components of the basicranial landmark configuration that were significantly correlated with developmental age and centroid size: (a) PC1* versus* PC2 (representing 14.83% and 9.31% of the variance, resp.); (b) PC3* versus* PC4 (7.57% and 6.53% variance, resp.). Each quadrant of the plot contains a wireframe indicating the morphology typical for the specimens in that quadrant.

The regression of biological age with Principal Components scores (Tables 14 and 15) for the entire basicranium revealed significant correlations between age and PC1 (14.83% of the variance), PC2 (9.31% variance), and PC4 (6.53% variance) (Figures 2 and 3). While these PCs each describe a relatively low amount of variance, this pattern is typical in PCAs following a Procrustes superimposition, because the Procrustes analysis essentially removes the effect of size. PC1 was found to be correlated with age for all populations for morphology of both the entire basicranium and the isolated temporal bone (Table 15). Additionally, PC4 was correlated with biological age for sphenoid morphology in all individual populations except the Polynesians, and PC6 was correlated with age for the occipital bone in all individual populations except the Peruvian sample (Table 15).

#### 3.3.2. Centroid Size

The regression analysis of centroid size with PC scores for the entire basicranium revealed significant correlations between size and PC1 (14.83% of the variance), PC2 (9.31% of the variance), PC3 (7.57% of the variance), and PC4 (6.53% of the variance) (Tables 14 and 16). The regression plot of PC1 scores versus log centroid size is illustrated in Figure 2. PC1 was significantly correlated with centroid size for all the basicranial morphology of individual populations, except the Polynesian sample, and all individual populations for the temporal bone (Table 16), similar to the pattern revealed by the age versus PC1 comparisons (Table 15). This reflects the fact that size and age are related, as expected. PC3 was significantly correlated with centroid size for all individual populations in the occipital bone, a pattern not seen in all of the various age categories. Similar to the age comparisons, the centroid size of the sphenoid bone and PC4 were significantly correlated for all populations individually. Mean PC scores for each age group in each population are provided for comparative purposes (Tables 17 and 18).

### 3.4. Shape Space Exploration

In order to further explore how populations and age categories differed with regard to basicranial morphology, we conducted a shape space exploration in which wireframes of each cranial data set were morphed along the major PC axes to visualize how the shape varied along each PC.

In basicranial shape, subadults were commonly located on the +PC1 axis (Figure 3(a)). A positive PC1 score is associated a mediolaterally narrow basicranium, with an elongated external acoustic meatus (EAM). The external occipital protuberance (EOP) also moves relatively further away from the occipital condyle with an increasingly positive PC1 score. Given that most of the subadult values appeared on the +PC1 side, this indicates that the subadult basicranium is longer and narrower but widens into adulthood, while relatively decreasing in length. A negative PC1 score is characterized by the reverse morphological conditions (i.e., mediolaterally wider basicranial, shortened EAM, closer proximity of the EOP, and occipital condyle).

Associated with a positive PC2 score (Figure 3(a)), the EAM becomes relatively larger, but the overall basicranial length decreases. The hypoglossal canal and articular eminence move relatively further posteriorly. The length between the occipital condyle and the EOP decreases as well, thus shortening the basicranium. The EAM length relatively increases.

## 4. Discussion

To date, extensive research has been conducted on the temporal bone and how it reflects genetic relationships in nonhuman primates (e.g., [13, 14, 31–33, 48]). In modern humans, it has been determined that the temporal bone reflects genetic relationships in both subadults and adults and that many populations significantly differ in their ontogenetic trajectories [7]. The current study focused on a larger area of the skull, the basicranium and its constituent regions, and their ontogenetic patterns.

### 4.1. Hypotheses

The hypotheses that formed the basis for the study were found to be only partially supported. In actuality, the patterns of morphological variation turned out to be more complex than the relatively simply stated hypotheses. The patterns of the individual basicranial bones differed from each other and across the various age categories. Thus, the ontogenetic processes driving basicranial development cannot be succinctly summarized across all regions of the basicranium.

(H1) Human populations differ in the shape of the basicranium and its various components irrespective of ontogenetic stage.

This hypothesis was not supported by our results. All populations were found to differ significantly in basicranial morphology in the combined subadult samples. However, in the separate age cateories there was a trend toward increasing differentiation with age. In AC1, several population were not significantly different, by AC2 most populations differed, and by AC3 all populations were significantly different. Interestingly, of all the individual cranial bones, the sphenoid was revealed to be the most distinct among populations and therefore the most reliable region for population classification.

(H2) Differences among human populations in basicranial morphology are significantly correlated with their genetic distances throughout ontogeny.

This hypothesis was partially supported for several subsets of our data. Our results show that morphology of the basicranium, occipital, and temporal bones each significantly reflects genetic distances in the combined subadult sample. The sphenoid bone, however, is not a good indicator of genetic distances in subadults in the combined subadult sample or at any ontogenetic stage. In the separate age categories for the basicranium, temporal, and occipital regions, the older the individuals, the more congruent the patterns between the genetic and morphological datasets, such that AC3 was the most highly correlated, followed by AC2 and then AC1.

### 4.2. Classification among Populations

The discriminant function analyses revealed that the Egyptian population was the most correctly classified population for the basicranium, occipital, and temporal bone in subadults. The Egyptian population was most likely classified correctly because it is the most geographically and genetically distant of the populations in this study. This finding is consistent with the previous description of Egyptian cranial morphology as unique and distinctive [49].

Unsurprisingly, the closely related Native American groups of Utah and Mexico were often classified as each other in basicranial, temporal, and occipital bone for all subadults. This trend has been supported by previous studies [6, 7] and supports the hypothesis that closely related populations should share a similar basicranial shape. Relethford [6] showed that the Peruvian population was phenotypically very similar to other Native American populations in his study (Arikara, Greenland Inuit and Santa Cruz) despite its remote geographic location compared to the other Native American populations. Consistent with the results of this study, Smith and colleagues [7] also found that the temporal bone of the subjects from Utah and Mexico was most often classified as another Native American population. In fact, the low levels of correct classification for these populations suggest that their morphological variation overlaps considerably in the younger age categories. These findings, taken together, show that the basicranial morphology can generally be used as an indicator of genetic relatedness among children and adolescents but considerable overlap may still exist for populations which are close in genetic proximity, such as Native American populations.

Based on the findings of this research, the basicranium, temporal, and occipital bone reflect genetic distances in childhood and adolescence, but this study suggests that these differences are not seen in infancy. AC1 was not correlated with molecular distances among populations for any of the cranial regions. Two possible scenarios may explain these results in infancy. First, infant basicranial morphology may be similar among populations and the observable differences in later ontogenetic stages have not yet developed. However, an alternative explanation is that as a result of the fact that many basicranial bones are not fully fused in infants, our sample sizes in the AC1 age category may have been insufficient to reveal subtle differences among populations in infancy.

These findings contrast with some of the results found previously for the temporal bone [7, 31], which suggest that the relationships in temporal bone originate early in ontogeny and these differences reach adulthood via different ontogenetic trajectories. Terhune et al. [31] also found that subadult great apes and humans demonstrate differences in temporal bone shape early in ontogeny. Contrary to Smith et al. [7] and Terhune et al. [31] for the temporal bone, and Viðarsdóttir et al. [8] for the face, the present study did not find that early subadult morphology (i.e., AC1) reflects adult population-specific differences. This finding is surprising given that the temporal bone has been found previously to reflect genetic distances in adult humans [2–7]. However, these seemingly contrasting results may reflect the choice of different landmarks used in the present study. Alternatively, these differences may be explained by the relatively small sample sizes necessitated by the limited subadult cranial material available in museum collections.

Interestingly, while morphology of the sphenoid was not found to reflect the molecular distance matrix at any ontogenetic stage evaluated (Table 9), it did perform quite well in the DFAs (Table 8). Thus, the morphology of this bone can be inferred to be distinct among the populations sampled here; however, those differences do not reflect genetic relatedness. In other words, more closely related populations do not share similar sphenoid morphology, but instead, each population is unique in its sphenoid shape.

### 4.3. Cranial Morphology and Genetic Relationships

Overall patterns revealed by this study show that the shape of the basicranium, the temporal bone, and occipital bone, for all populations excluding the Egyptian population, reflects genetic distances in subadults. Removing the Egyptian population from the analyses yielded significant results in the temporal bone, occipital bone, and age categories AC2 and AC3. It cannot be definitively ascertained why the Egyptian population deviated from the patterns of the rest of the included samples, but one possibility is the relatively high degree of mismatch between the morphological Egyptian sample and its molecular population representative, the Mozabite. The Mozabite people live in Algeria and speak a Berber language. As one of the few northern Saharan populations to have been extensively studied for neutral molecular loci, they are the closest well-typed molecular representative for the Egyptians, but certainly not a perfect match.

Patterns on the basicranial PC wireframe showed that the Egyptian crania appeared on the +PC2 scale, indicating that the basicranium in the Egyptian population is relatively shorter anteroposteriorly compared to the other populations. Most of the Egyptian population clustered towards an increasingly long occipital bone (–PC2, +PC3, and –PC4) and increasing large temporal bone. It appears that the Egyptian cranium might have a relatively smaller overall basicranium but that the temporal and occipital bone increased in length, with the sphenoid decreasing in length to compromise.

A study by A. C. Berry and R. J. Berry [49] found that the Egyptian population was characterized by distinctive cranial morphology, which had persisted for centuries and was dissimilar to the crania of other populations in their study. Based on the current findings, the Egyptian population showed a relatively decreasing anteroposterior length but increasingly elongated temporal and occipital bones compared to other population samples in this study. Given its genetic and geographic distance and cranial stability, the Egyptian population could have likely acted as a morphological and genetic outlier, and only with its exclusion did the results begin to show significant differences among populations in various regions of the basicranium and age categories.

One potentially limiting factor of this study is the degree of mismatch between the molecular and morphological population representatives. The morphological samples were chosen based on the availability of subadult cranial material in museum collections. Thus, we were not able to include a wide geographically distribution of populations. In North American natural history collections, there is a natural bias towards Native American specimens. Consequently, the present study included a few Native American samples. Similarly, the molecular representative for each population was not a perfect match with these morphological populations. However, there is precedent for this approach. As noted by Roseman (2004), such mismatch is not necessarily problematic, but the correlations obtained from this type of analysis should be interpreted as minimal approximations of their actual value. Thus, the significant correlations obtained here should be interpreted as minimum estimates of a real biological relationship between morphology of the cranial region and genetic relatedness.

The landmark dataset employed in the present research expands the smaller landmark sets used by previous studies. Harvati and Weaver [2] and Smith et al. [34] each used a much smaller and less anatomically distributed set of landmarks, 13 and 15, respectively, while our study included 24. There were also some differences in landmark coverage and dispersion between this and prior studies. The temporal bone landmarks in this study were widely distributed to include points that overlap with the sphenoidal and occipital regions of the basicranium, covering a large area of all components of the basicranium. We used landmarks distributed from the asterion in the occipital region and auriculare in the temporal region, to the suture between the temporal and zygomatic bones, for a total of 44 landmarks. Harvati and Weaver [2] also used several landmarks on the petrotympanic crest, which this study did not include. Smith et al. [34] included landmarks on the mandibular fossa, porion, and entoglenoid process, which were not used in this study.

Occipital bone morphology is valuable for assessing relatedness in adolescents starting around the age of 13 years old. The sphenoid bone, on the other hand, does not reflect genetic relatedness at any ontogenetic stage; however, despite this, the morphology is distinct enough among populations to allow unidentified specimens to be classified by population with rather high accuracy. This is likely because the sphenoid body of basicranium reaches adult size and shape more rapidly than other portions, presumably because vital cranial nerves (II–VI) run through the cranial base in the sphenoid region [13, 16]. This may also result in a more constrained range of variation, since the sphenoid housed these essential neurological structures.

Population relatedness can be inferred using the basicranium, temporal, and occipital bone of subadults, especially those of 13–18 years of age. The findings of this study have implications for future studies of archaeological specimens for which genetic material is not well-preserved. Given that the morphology of the temporal bone and basicranium reflect genetic distances in young subadults, the morphology of these cranial subsets can be used to sort human populations with a reasonable degree of precision (25% and 41% mean classification, resp.). This could be useful for child or adolescent cranial specimens of unknown affinity found at archaeological sites.

If future studies examine other hominoid (ape) species in a similar manner, the findings combined with hose of the present study would have implications for the development of hominoid brain size, posture, and evolution. First, the angle of the midline cranial base is hypothesized to correlate with the volume of the brain relative to its basicranial length [50–52]. Thus, findings relating to how the basicranium changes during ontogeny can contribute to a more comprehensive understanding of brain development in various ape species. Second, flexion of the cranial base has been interpreted to be an adaptation for upright posture in hominins, causing the foramen magnum to position anteriorly and orient ventrally [53–55]. The basicranium therefore affects the verticality of hominin posture. Third, the basicranium likely played a role in the evolution of the overall morphology of the primate skull [14, 23]. Specifically, the cranial base develops early in ontogeny and thus influences the development of later-ossifying regions, such as the facial skeleton and neurocranium [14, 23]. Evolutionary changes in the basicranium will necessarily impact the adaptive morphology of the entire primate cranial apparatus.

## 5. Conclusion

In this study, the basicranium, occipital, and temporal regions were found to reflect genetic distances among populations in childhood and adolescence. The sphenoid bone, however, is not a good indicator of genetic distances in subadults, but its morphology may still serve as a reasonably accurate means of classifying individuals by population affinity. Unsurprisingly, the Native American populations, and especially the Utah sample, were commonly classified as another Native American population, as would be predicted if basicranial shape reflects genetic relatedness. These findings reveal valuable information on the population differences in basicranial morphology at various ontogenetic stages.

## Figures and Tables

**Table 1:** Human population samples, their adult and subadult sample sizes, and molecular representative populations.

Population	AC1 (*n*)	AC2 (*n*)	AC3 (*n*)	Total (*n*)	Molecular representative
Alaskan	10	6	14	30	Yakut
Austrian	5	10	13	28	French
Egyptian	9	7	11	27	Mozabite
Mexican	8	7	15	30	Maya
Peruvian	3	8	19	30	Colombians
Polynesian	2	6	9	17	Solomon Islanders
Utah Native American	11	7	4	22	Pima
Total	**48**	**51**	**85**	**184**	

**(a):** 

Occipital	
1	Most anterior point on the basioccipital in the midline (sphenobasion, on the occipital if not connected)^*∗*^
2	Most lateral point on the basioccipital^*∗*^
3	Basion (anterior most point on the foramen magnum)
4	Most anterior point on the occipital condyle along the margin of the foramen magnum
5	Most anterior point on occipital condyle
6	Most lateral point on the occipital condyle (point on the middle of the lateral edge of the condyle)
7	Most posterolateral point on the occipital condyle
8	Most posterior point on the occipital condyle along the margin of the foramen magnum
9	Mid-point of the occipital condyle (inferior aspect)
10	Opisthion (posterior most point on the foramen magnum)
11	Mid-point on the median nuchal line between the external occipital protuberance and foramen magnum
12	Anteromedial point on the hypoglossal canal
13	Asterion (temporal, occipital, and parietal meet)^*∗*^

^*∗*^Landmarks are repeated with regard to the overlapping cranial regions.

**(b):** 

Temporal	
13	Asterion (where temporal, occipital, and parietal meet)^*∗*^
14	Parietal notch (not depicted)
15	Mastoidale (center of the inferior point on the mastoid process)
16	Most lateral point on the margin of the stylomastoid foramen
17	Most lateral point on the vagina of the styloid process (whether process is present or absent)
18	Most posterolateral point on the jugular fossa
19	Most posterolateral point on the margin of the carotid canal entrance
20	Point on anterior margin of tympanic element that is closest to carotid canal
21	Most posterolateral point on the external acoustic meatus
22	Most inferior point on the external acoustic meatus
23	Point on lateral margin of zygomatic process of the temporal bone at the position of the postglenoid process
24	Point of inflection where the braincase curves laterally into the supraglenoid gutter, in coronal plane of mandibular fossa (not depicted)
25	Point on the anterior of the lateral margin of the articular surface of the articular eminence
26	Most inferior point on the postglenoid process
27	Deepest point within the mandibular fossa (instrumentally determined)
28	Mid-point of the articular eminence
29	Most anterior point on the articular surface of the articular eminence
30	Auriculare (most lateral point on the temporal)
31	Suture between temporal and zygomatic bones on inferior aspect of zygomatic process
32	Most inferior point at the sphenotemporal suture closer to the midline (on the sphenoid if disconnected)
33	Most lateral point on the greater wing of the sphenoid (intersection between sphenoid, temporal, and parietal bone)^*∗*^ (not depicted)
34	Most frontolateral point on the greater wing of the sphenoid (intersection between sphenoid, temporal, and frontal bone)^*∗*^ (not depicted)
36	Most posterior, inferior point on the sphenotemporal suture^*∗*^
37	Apex of the petrous part of the temporal bone

^*∗*^Landmarks are repeated with regard to the overlapping cranial regions.

**(c):** 

Sphenoid	
33	Most lateral point on the greater wing of the sphenoid (intersection between sphenoid, temporal, and parietal bone)^*∗*^ (not depicted)
35	Most anterior inferior point on the sphenozygomatic suture (sphenozygomatic) (not depicted)
36	Most posterior, inferior point on the sphenotemporal suture^*∗*^
38	Most lateral point of the foramen spinosum
39	Most lateral point on the margin of foramen ovale
40	Most anterolateral point of the lateral pterygoid plate
41	Most inferior part of the pterygoid hamulus (not depicted)
42	The most anteromedial point of the sphenoidal region on the sphenovomer suture
43	Most posterior point where the vomer meets the medial pterygoid plate
44	Point on the sphenoid in the midline in contact with the vomer (vomer notch)
1	Most anterior point on the basioccipital in the midline (sphenobasion, on the occipital if not connected)^*∗*^
2	Most lateral point on the basioccipital^*∗*^

^*∗*^Landmarks are repeated with regard to the overlapping cranial regions.

**Table 3:** Population molecular distance matrix (*F*_ST_) based on short tandem repeat (STR) data. Data were obtained from Rosenberg et al. (2005) [35].

	Alaska	Austria	Egypt	Mexico	Peru	Polynesia	Utah Native American
Alaska	—						
Austria	0.0455	—					
Egypt	0.0530	0.1068	—				
Mexico	0.0580	0.0606	0.0731	—			
Peru	0.0951	0.0984	0.1110	0.0443	—		
Polynesia	0.0759	0.0759	0.0808	0.1028	0.1443	—	
Utah Native American	0.0984	0.1076	0.1190	0.0600	0.0973	0.1461	—

**Table 4:** Procrustes distance matrix among populations based on subadult basicranial morphology. All pairwise population distances are significantly different at the *p* < 0.05 level.

	Alaska	Austria	Egypt	Mexico	Peru	Polynesia	Utah
Alaska	—						
Austria	0.0361	—					
Egypt	0.0779	0.0703	—				
Mexico	0.0398	0.0444	0.0745	—			
Peru	0.0493	0.0513	0.0846	0.0411	—		
Polynesia	0.0618	0.0532	0.0809	0.0631	0.0740	—	
Utah	0.0694	0.0684	0.0998	0.0638	0.0548	0.0805	—

**Table 5:** Morphological Procrustes distance matrix among populations based on basicranial morphology in the AC1 age category. Significantly different pairwise population distances (*p* < 0.05) are indicated in bold.

	Alaska	Austria	Egypt	Mexico	Peru	Polynesia	Utah
Alaska	—						
Austria	**0.1580**	—					
Egypt	0.1110	0.1611	—				
Mexico	**0.1341**	0.1518	0.0816	—			
Peru	0.1127	0.1867	**0.1581**	0.1571	—		
Polynesia	0.1045	0.1735	0.1066	0.1102	0.1519	—	
Utah	**0.1068**	0.1485	0.1040	0.0908	**0.1592**	0.1019	—

**Table 6:** Morphological Procrustes distance matrix among populations based on basicranial morphology in the AC2 age category. Significantly different pairwise population distances (*p* < 0.05) are indicated in bold.

	Alaska	Austria	Egypt	Mexico	Peru	Polynesia	Utah
Alaska	—						
Austria	**0.0870**	—					
Egypt	**0.1289**	**0.1337**	—				
Mexico	**0.0831**	**0.0765**	0.0948	—			
Peru	**0.1035**	**0.0867**	**0.1423**	0.0799	—		
Polynesia	0.0799	**0.0940**	**0.1016**	**0.0849**	**0.1115**	—	
Utah	**0.0888**	**0.0869**	**0.1039**	0.0722	**0.0871**	0.0715	—

**Table 7:** Morphological Procrustes distance matrix among populations based on basicranial morphology in the AC3 age category. Significantly different pairwise population distances (*p* < 0.05) are indicated in bold.

	Alaska	Austria	Egypt	Mexico	Peru	Polynesia	Utah
Alaska	—						
Austria	**0.0543**	—					
Egypt	**0.0994**	**0.0921**	—				
Mexico	**0.0559**	**0.0601**	**0.0909**	—			
Peru	**0.0558**	**0.0682**	**0.1021**	**0.0506**	—		
Polynesia	**0.0659**	0.0620	**0.1031**	**0.0774**	**0.0796**	—	
Utah	**0.0953**	**0.0960**	0.1382	**0.0886**	0.0795	**0.1042**	—

**Table 8:** Classification results from discriminant function analysis (DFA) with cross-validation for the entire basicranium.

	% Correct	Alaska	Austria	Egypt	Mexico	Peru	Polynesia	Utah	Total
Alaska	26.3	5	2	0	2	3	4	3	19
Austria	34.8	3	8	1	6	4	1	0	23
Egypt	33.3	0	0	3	1	1	3	1	9
Mexico	13.3	3	3	1	2	3	1	2	15
Peru	20.8	5	4	1	5	5	1	3	24
Polynesia	20.0	2	0	4	0	1	2	1	10
Utah	28.6	1	0	0	2	2	0	2	7

25.2% of cross-validated grouped cases correctly classified.

**Table 9:** Classification results from discriminant function analysis (DFA) with cross-validation for the temporal bone.

	% Correct	Alaska	Austria	Egypt	Mexico	Peru	Polynesia	Utah	Total
Alaska	65.4	17	2	0	3	0	0	4	26
Austria	37.0	7	10	1	4	4	0	1	27
Egypt	55.0	1	4	11	2	1	1	0	20
Mexico	23.1	2	5	2	6	6	1	4	26
Peru	48.3	2	5	1	3	14	0	4	29
Polynesia	42.9	2	2	2	1	1	6	0	14
Utah	11.1	5	1	1	4	4	1	2	18

41.3% of cross-validated grouped cases correctly classified.

**Table 10:** Classification results from discriminant function analysis (DFA) with cross-validation for the occipital bone.

	% Correct	Alaska	Austria	Egypt	Mexico	Peru	Polynesia	Utah	Total
Alaska	30	6	7	0	3	3	1	NA	20
Austria	33.3	7	8	5	2	0	2	NA	24
Egypt	35.7	1	5	5	1	1	1	NA	14
Mexico	31.3	3	2	2	5	4	0	NA	16
Peru	75	2	0	1	3	18	0	NA	24
Polynesia	40	1	2	2	1	0	4	NA	10
Utah	NA	NA	NA	NA	NA	NA	NA	NA	NA

42.6% of cross-validated grouped cases correctly classified.

**Table 11:** Classification results from discriminant function analysis (DFA) with cross-validation for sphenoid morphology.

	% Correct	Alaska	Austria	Egypt	Mexico	Peru	Polynesia	Utah	Total
Alaska	41.2	7	5	0	0	3	0	2	17
Austria	55.0	3	11	2	2	1	0	1	20
Egypt	30.0	0	3	3	1	2	0	1	10
Mexico	57.1	2	0	0	8	4	0	0	14
Peru	63.6	2	2	1	2	14	0	1	22
Polynesia	42.9	0	0	1	1	2	3	0	7
Utah	37.5	2	2	0	1	0	0	3	8

50.0% of cross-validated grouped cases correctly classified.

**Table 12:** Procrustes analyses comparing molecular distances with morphological distances based on each of the cranial data sets.

	Basicranium		Temporal		Occipital		Sphenoid	
	*R*	*p* value	*R*	*p* value	*R*	*p* value	*R*	*p* value
All subadults	**0.48**	**0.04** ^*∗*^	0.18	0.30	**0.82**	**<0.001** ^*∗*^	0.33	0.14
AC1	−**0.58**	**<0.001** ^*∗*^	−0.38	0.09	NA	NA	NA	NA
AC2	0.36	0.06	−0.18	0.28	**0.58**	**0.02** ^*∗*^	0.09	0.38
AC3	0.33	0.18	0.14	0.34	**0.84**	**<0.001** ^*∗*^	−0.06	0.56

^*∗*^Statistically significant (*p* < 0.05).

**Table 13:** Procrustes analyses comparing molecular distances with morphological distances based on each of the cranial data sets excluding the Egyptian population.

	Basicranium		Temporal		Occipital		Sphenoid	
	*R*	*p* value	*R*	*p* value	*R*	*p* value	*R*	*p* value
All subadults	**0.83**	**<0.001** ^*∗*^	**0.79**	**<0.001** ^*∗*^	**0.88**	**0.01** ^*∗*^	0.55	0.05
AC1	−0.45	0.17	−0.15	0.36	NA	NA	NA	NA
AC2	**0.57**	**<0.001** ^*∗*^	**0.46**	**<0.001** ^*∗*^	−0.01	0.50	0.09	0.38
AC3	**0.91**	**<0.001** ^*∗*^	**0.81**	**0.01** ^*∗*^	**0.91**	**<0.001** ^*∗*^	−0.26	0.22

^*∗*^Statistically significant (*p* < 0.05).

**Table 14:** Principal Components of the basicranium of all populations significantly correlated with age and/or centroid size in the regression analysis.

	Age	Centroid size
PC1	*R* = −0.63, *p* < 0.001^*∗*^	*R* = −0.50, *p* < 0.001^*∗*^
PC2	*R* = 0.29, *p* < 0.001^*∗*^	*R* = 0.26, *p* < 0.001^*∗*^
PC3	*R* = 0.02, *p* = 0.40	*R* = 0.14, *p* = 0.03^*∗*^
PC4	*R* = 0.12, *p* = 0.04^*∗*^	*R* = 0.14, *p* = 0.03^*∗*^

^*∗*^Statistically significant (*p* < 0.05).

**Table 15:** Principal Components of the basicranium and its regions significantly correlated with age in the regression analysis.

	Basicranium	Temporal	Occipital	Sphenoid
All populations	PC1, PC2, PC4	PC1, PC2, PC4	PC3, PC5, PC6	PC2, PC4
Alaska	PC1, PC2, PC4	PC1, PC4	PC2, PC3, PC4, PC6	PC1, PC2, PC4
Austria	PC1, PC3, PC4	PC1, PC2, PC3, PC6	PC1, PC2, PC6	PC4
Egypt	PC1	PC1	PC6	PC4
Mexico	PC1	PC1, PC2	PC3, PC4, PC6	PC4
Peru	PC1, PC2, PC3	PC1, PC6	PC1, PC2, PC5	PC4
Polynesia	PC1	PC1	PC3, PC5, PC6	
Utah	PC1, PC2	PC1, PC2	PC5, PC6	PC2, PC4

**Table 16:** Principal Components of the basicranium and its regions significantly correlated with centroid size in the regression analysis.

	Basicranium	Temporal	Occipital	Sphenoid
All populations	PC1, PC2, PC3, PC4	PC1, PC4, PC6	PC1, PC2, PC3, PC4, PC6	PC1, PC2, PC4
Alaska	PC1, PC2	PC1, PC4	PC2, PC3, PC4, PC6	PC1, PC4
Austria	PC1, PC3, PC4	PC1, PC2, PC4, PC6	PC3	PC2, PC4, PC5
Egypt	PC1, PC4, PC5	PC1, PC4	PC1, PC3	PC1, PC3, PC4, PC5
Mexico	PC1, PC2, PC4	PC1, PC3, PC4	PC1, PC2, PC3, PC4, PC5, PC6	PC4
Peru	PC1, PC2	PC1, PC4	PC1, PC3	PC4
Polynesia		PC1, PC2	PC2, PC3	PC2, PC4
Utah	PC1, PC2	PC1	PC1, PC3	PC1, PC4

**Table 17:** Mean Principal Components scores for the major PCs for each age group in each population for the entire basicranium. Note: sample size of complete basicrania of AC1 for the Polynesian population was insufficient to obtain a reasonable estimation of the mean.

Population	Age Cat.	PC1	PC2	PC3	PC4	PC5
Alaskans	AC1	0.05323	0.00302	0.00175	−0.00882	0.00855
Alaskans	AC2	0.04618	−0.01399	0.01446	−0.01085	−0.00150
Alaskans	AC3	0.02569	0.00815	−0.00014	−0.01146	0.00114
Alaskans	Adults	0.00969	0.00543	0.00499	−0.00409	0.00546
Austrians	AC1	0.07867	−0.02117	−0.03936	−0.03364	0.00580
Austrians	AC2	0.02695	0.00316	0.00068	0.02413	0.01203
Austrians	AC3	0.00584	0.02062	0.00347	0.02083	−0.00779
Austrians	Adults	−0.02377	−0.01075	0.00784	0.02859	0.00468
Egyptians	AC1	0.11390	0.02214	0.00609	−0.01172	0.00766
Egyptians	AC2	0.04530	−0.00072	−0.00789	0.00435	−0.02901
Egyptians	AC3	0.00920	0.01193	−0.00507	0.03377	−0.05306
Egyptians	Adults	0.00662	0.03986	−0.00195	0.01716	−0.01972
Mexicans	AC1	0.08308	0.02326	−0.02515	−0.01663	−0.01222
Mexicans	AC2	0.02838	−0.02994	0.01315	0.01064	0.00299
Mexicans	AC3	0.01160	−0.02266	−0.01056	0.00150	0.00459
Mexicans	Adults	−0.03151	−0.00378	0.00344	−0.00451	0.00588
Peruvians	AC1	0.00004	−0.10173	0.02027	0.01840	0.00450
Peruvians	AC2	−0.01429	−0.04447	0.00779	−0.00991	0.01923
Peruvians	AC3	−0.01735	−0.03330	0.01130	−0.01073	−0.00269
Peruvians	Adults	−0.03927	0.00426	−0.00411	−0.00933	0.00495
Polynesians	AC1	—	—	—	—	—
Polynesians	AC2	0.06510	0.02825	−0.00041	−0.00531	−0.00052
Polynesians	AC3	0.01269	0.04162	0.00118	0.00674	0.00423
Polynesians	Adults	−0.05657	0.04434	−0.01331	0.01068	0.00484
Utah	AC1	0.05701	−0.05851	−0.00487	−0.03191	0.01686
Utah	AC2	0.00952	−0.01100	−0.00275	−0.03556	−0.00031
Utah	AC3	−0.06937	−0.00900	−0.00478	−0.02770	0.00412
Utah	Adults	−0.04365	0.00478	−0.00146	−0.02958	−0.00178

**Table 18:** Mean Principal Components scores for the major PCs for each age group in each population for the temporal bone.

Population	Age Cat.	PC1	PC2	PC3	PC4	PC5	PC6
Alaskans	AC1	0.06370	0.03778	−0.00467	0.003486	−0.01318	0.002793
Alaskans	AC2	0.05955	0.01832	−0.01152	0.012063	−0.00951	0.006615
Alaskans	AC3	0.01364	0.01492	−0.01702	−0.00626	0.01089	−0.00095
Alaskans	Adults	−0.04098	0.00911	−0.03013	−0.01553	0.003458	−0.00611
Austrians	AC1	0.06432	0.04918	0.014509	0.0147	0.004334	0.041771
Austrians	AC2	0.01920	−0.01637	−0.01942	−0.00831	−0.02196	0.000631
Austrians	AC3	−0.00658	−0.00682	−0.00317	−0.0133	−0.00137	−0.00585
Austrians	Adults	−0.04079	−0.01804	0.036348	−0.00234	−0.01313	−0.01497
Egyptians	AC1	0.09614	−0.01266	−0.00793	−0.00211	0.011929	0.021407
Egyptians	AC2	0.07456	−0.07440	0.023618	0.005589	0.039431	0.00116
Egyptians	AC3	0.03027	−0.01573	−0.01638	−0.02419	−0.01115	0.008317
Egyptians	Adults	−0.00847	−0.01948	−0.0062	−0.01665	0.009855	0.022317
Mexicans	AC1	0.07394	0.03163	−0.00879	0.015592	0.024777	0.01061
Mexicans	AC2	0.04101	0.00014	−0.0111	0.009107	0.003618	−0.02052
Mexicans	AC3	−0.00346	0.00340	−0.01835	0.013748	0.007935	−0.00232
Mexicans	Adults	−0.02252	−0.03734	0.005864	−0.02859	0.023767	0.001946
Peruvians	AC1	0.04345	−0.01427	0.00188	0.00293	0.031773	−0.00416
Peruvians	AC2	0.01024	0.00996	0.006532	0.025246	−0.00551	−0.01473
Peruvians	AC3	−0.01153	0.01216	0.011737	0.022836	−0.00735	−0.0178
Peruvians	Adults	−0.04831	−0.00314	0.015741	0.009016	−0.00291	0.010455
Polynesians	AC1	0.09089	0.03686	0.01485	−0.01808	−0.08961	−0.02056
Polynesians	AC2	0.03138	0.04798	−0.01105	−0.00197	0.01271	0.000566
Polynesians	AC3	−0.01459	−0.01922	0.003433	−0.01135	−0.00779	0.009155
Polynesians	Adults	−0.07627	−0.00224	0.001625	−0.01349	−0.00344	0.022897
Utah	AC1	0.10426	−0.00679	0.01661	0.030121	−0.0139	−0.01324
Utah	AC2	0.04445	−0.00898	0.018823	0.00818	−0.00028	0.003031
Utah	AC3	−0.05003	0.02901	0.014978	−0.01203	0.021723	−0.00859
Utah	Adults	−0.04109	0.00599	0.030287	0.003773	0.000573	−0.01027

## References

[1] Roseman C. C. (2004). Detecting interregionally diversifying natural selection on modern human cranial form by using matched molecular and morphometric data. *Proceedings of the National Academy of Sciences of the United States of America*.

[2] Harvati K., Weaver T. D. (2006). Human cranial anatomy and the differential preservation of population history and climate signatures. *The Anatomical Record A*.

[3] Smith H. F. (2009). Which cranial regions reflect molecular distances reliably in humans? Evidence from three-dimensional morphology. *The American Journal of Human Biology*.

[4] von Cramon-Taubadel N. (2009). Congruence of individual cranial bone morphology and neutral molecular affinity patterns in modern humans. *American Journal of Physical Anthropology*.

[5] von Cramon-Taubadel N. (2009). Revisiting the homoiology hypothesis: the impact of phenotypic plasticity on the reconstruction of human population history from craniometric data. *Journal of Human Evolution*.

[6] Relethford J. H. (2010). Population-specific deviations of global human craniometric variation from a neutral model. *American Journal of Physical Anthropology*.

[7] Smith H. F., Ritzman T., Otárola-Castillo E., Terhune C. E. (2013). A 3-D geometric morphometric study of intraspecific variation in the ontogeny of the temporal bone in modern *Homo sapiens*. *Journal of Human Evolution*.

[8] Viðarsdóttir U. S., O'Higgins P., Stringer C. (2002). A geometric morphometric study of regional differences in the ontogeny of the modern human facial skeleton. *Journal of Anatomy*.

[9] Vidarsdottir U. S., O'Higgins P., Thompson J. L., Krovitz G. E., Nelson A. J. (2003). Development variation in the facial skeleton of anatomically modern *Homo sapiens*. *Patterns of Growth and Development in the Genus Homo*.

[10] Cobb S. N., O'Higgins P. (2004). Hominins do not share a common postnatal facial ontogenetic shape trajectory. *Journal of Experimental Zoology, Part B: Molecular and Developmental Evolution*.

[11] Olson T. R., Stringer C. B. (1981). Basicranial morphology of the extant hominoids and Pliocene hominids: the new material from the Hadar Formation, Ethiopia and its significance in early human evolution and taxonomy. *Aspects of Human Evolution*.

[12] Houghton P. (1996). *The People of the Great Ocean: Aspects of Human Biology in the Early Pacific*.

[13] Lieberman D. E., Ross C. F., Ravosa M. J. (2000). The primate cranial base: ontogeny, function, and integration. *American Journal of Physical Anthropology*.

[14] Lieberman D. E., Pearson O. M., Mowbray K. M. (2000). Basicranial influence on overall cranial shape. *Journal of Human Evolution*.

[15] Harvati K. (2001). *The Neanderthal problem: 3-D geometric morphometric models of cranial shape variation within and among species [Ph.D. thesis]*.

[16] Sperber G. H. (1981). *Craniofacial Embryology*.

[17] de Beer G. (1985). *The Development of the Vertebrate Skull*.

[18] Jeffery N., Spoor F. (2004). Ossification and midline shape changes of the human fetal cranial base. *American Journal of Physical Anthropology*.

[19] Wood B. A., Grine F. E. (1988). Are the ‘robust’ australopithecines a monophyletic group?. *Evolutionary History of the “Robust” Australopithecines*.

[20] Skelton R. R., McHenry M. (1992). Evolutionary relationships among early hominids. *Journal of Human Evolution*.

[21] Turner A., Wood B. A. (1993). Comparative palaeontological context for the evolution of the early hominid masticatory system. *Journal of Human Evolution*.

[22] Mchenry H. M. (1994). Tempo and mode in human evolution. *Proceedings of the National Academy of Sciences of the United States of America*.

[23] Lieberman D. E., Wood B. A., Pilbeam D. R. (1996). Homoplasy and early *Homo*: an analysis of the evolutionary relationships of *H. habilis sensu stricto* and *H. rudolfensis*. *Journal of Human Evolution*.

[24] Moore W. J., Lavelle C. J. B. (1974). *Growth of the Facial Skeleton in the Hominoidea*.

[25] MacPhee R. D. E., Cartmill M., Swindler D. R., Erwin J. (1986). Basicranial structures and primate systematics. *Comparative Primate Biology, Vol. 1: Systematics, Evolution, and Anatomy*.

[26] Wood B., Lieberman D. E. (2001). Craniodental variation in *Paranthropus boisei*: a developmental and functional perspective. *The American Journal of Physical Anthropology*.

[27] Lycett S. J., Collard M. (2005). Do homoiologies impede phylogenetic analyses of the fossil hominids? An assessment based on extant papionin craniodental morphology. *Journal of Human Evolution*.

[28] Collard M., Wood B. (2007). Hominin homoiology: an assessment of the impact of phenotypic plasticity on phylogenetic analyses of humans and their fossil relatives. *Journal of Human Evolution*.

[29] Eby T. L., Nadol J. B. (1986). Postnatal growth of the human temporal bone. Implications for cochlear implants in children. *Annals of Otology, Rhinology, and Laryngology*.

[30] Simms D. L., Neely J. G. (1989). Growth of the lateral surface of the temporal bone in children. *Laryngoscope*.

[31] Terhune C. E., Kimbel W. H., Lockwood C. A. (2013). Postnatal temporal bone ontogeny in *Pan, Gorilla*, and *Homo*, and the implications for temporal bone ontogeny in *Australopithecus afarensis*. *American Journal of Physical Anthropology*.

[32] Lockwood C. A., Lynch J. M., Kimbel W. H. (2002). Quantifying temporal bone morphology of great apes and humans: an approach using geometric morphometrics. *Journal of Anatomy*.

[33] Lockwood C. A., Kimbel W. H., Lynch J. M. (2004). Morphometrics and hominoid phylogeny: support for a chimpanzee-human clade and differentiation among great ape subspecies. *Proceedings of the National Academy of Sciences of the United States of America*.

[34] Smith H. F., Terhune C. E., Lockwood C. A. (2007). Genetic, geographic, and environmental correlates of human temporal bone variation. *American Journal of Physical Anthropology*.

[35] Rosenberg N. A., Mahajan S., Ramachandran S., Zhao C., Pritchard J. K., Feldman M. W. (2005). Clines, clusters, and the effect of study design on the inference of human population structure. *PLoS Genetics*.

[36] von Cramon-Taubadel N. (2011). The relative efficacy of functional and developmental cranial modules for reconstructing global human population history. *The American Journal of Physical Anthropology*.

[37] Ubelaker D. (1989). *Human Skeletal Remains: Excavation, Analysis, Interpretation*.

[38] White T. D., Folkens P. A. (2000). *Human Osteology*.

[39] Weber J. L., May P. E. (1989). Abundant class of human DNA polymorphisms which can be typed using the polymerase chain reaction. *American Journal of Human Genetics*.

[40] Ramachandran S., Deshpande O., Roseman C. C., Rosenberg N. A., Feldman M. W., Cavalli-Sforza L. L. (2005). Support from the relationship of genetic and geographic distance in human populations for a serial founder effect originating in Africa. *Proceedings of the National Academy of Sciences of the United States of America*.

[41] Shriver M. D., Jin L., Chakraborty R., Boerwinkle E. (1993). VNTR allele frequency distributions under the stepwise mutation model: a computer simulation approach. *Genetics*.

[42] Valdes A. M., Slatkin M., Freimer N. B. (1993). Allele frequencies at microsatellite loci: the stepwise mutation model revisited. *Genetics*.

[43] Goldstein D. B., Linares A. R., Cavalli-Sforza L. L., Feldman M. W. (1995). An evaluation of genetic distances for use with microsatellite loci. *Genetics*.

[44] Klingenberg C. P. (2011). MorphoJ: an integrated software package for geometric morphometrics. *Molecular Ecology Resources*.

[45] Excoffier L., Laval G., Schneider S. (2005). Arlequin (version 3.0): an integrated software package for population genetics data analysis. *Evolutionary Bioinformatics Online*.

[46] Mantel N. (1967). The detection of disease clustering and a generalized regression approach. *Cancer Research*.

[47] O'Higgins P., Jones N. (2006). *Tools for Statistical Shape Analysis*.

[48] Gilbert C. C., Frost S. R., Strait D. S. (2009). Allometry, sexual dimorphism, and phylogeny: a cladistic analysis of extant African papionins using craniodental data. *Journal of Human Evolution*.

[49] Berry A. C., Berry R. J. (1967). Epigenetic variation in the human cranium. *Journal of Anatomy*.

[50] DuBrul E. L., Laskin D. M. (1961). Preadaptive potentialities of the mammalian skull: an experiment in growth and form. *American Journal of Anatomy*.

[51] Gould S. J. (1977). *Ontogeny and Phylogeny*.

[52] Riesenfeld A. (1969). The adaptive mandible: an experimental study. *Acta Anatomica*.

[53] DuBrul E. L. (1977). Early hominid feeding mechanisms. *American Journal of Physical Anthropology*.

[54] DuBrul E. L., Sarnat B. G., Laskin D. M. (1979). Origin and adaptations of the hominid jaw joint. *The Temporomandibular Jaw Joint*.

[55] Schultz A. H. (1942). Conditions for balancing the head in primates. *American Journal of Physical Anthropology*.

